# Supplementary material for: Rtt105 regulates RPA function by configurationally stapling the flexible domains
Source: Nat Commun. 2022 Sep 2;13:5152. doi: 10.1038/s41467-022-32860-6 (PMC9440123; doi:10.1038/s41467-022-32860-6)
Supplement: Supplementary file 3 — Reporting Summary [file 41467_2022_32860_MOESM3_ESM.pdf]

nature portfolio

Corresponding author(s): Edwin Antony

Last updated by author(s): 08/03/2022

Reporting Summary

Nature Portfolio wishes to improve the reproducibility of the work that we publish. This form provides structure for consistency and transparency in reporting. For further information on Nature Portfolio policies, see our Editorial Policies and the Editorial Policy Checklist.

Statistics

For all statistical analyses, confirm that the following items are present in the figure legend, table legend, main text, or Methods section.

n/a	Confirmed	
☐	☒	The exact sample size (*n*) for each experimental group/condition, given as a discrete number and unit of measurement
☐	☒	A statement on whether measurements were taken from distinct samples or whether the same sample was measured repeatedly
☐	☒	The statistical test(s) used AND whether they are one- or two-sided<br>*Only common tests should be described solely by name; describe more complex techniques in the Methods section.*
☒	☐	A description of all covariates tested
☒	☐	A description of any assumptions or corrections, such as tests of normality and adjustment for multiple comparisons
☐	☒	A full description of the statistical parameters including central tendency (e.g. means) or other basic estimates (e.g. regression coefficient) AND variation (e.g. standard deviation) or associated estimates of uncertainty (e.g. confidence intervals)
☐	☒	For null hypothesis testing, the test statistic (e.g. F, t, r) with confidence intervals, effect sizes, degrees of freedom and P value noted<br>*Give P values as exact values whenever suitable.*
☒	☐	For Bayesian analysis, information on the choice of priors and Markov chain Monte Carlo settings
☒	☐	For hierarchical and complex designs, identification of the appropriate level for tests and full reporting of outcomes
☒	☐	Estimates of effect sizes (e.g. Cohen's *d*, Pearson's *r*), indicating how they were calculated

Our web collection on statistics for biologists contains articles on many of the points above.

Software and code

Policy information about availability of computer code

Data collection	Gels were scanned in the iBright-1500 imager (Thermo Fisher Scientific) and acquired using iBright software (ver.3.0.1).<br>Circular Dichroism data were collected on a Chirascan V100 instrument (Applied Photophysics Inc) using the associated Pro-Data Chirascan software (ver.4.7.0.194).<br>Steady-state fluorescence data were collected on an PC1 fluorimeter (ISS Inc.) using the instrument-associated Vinci 3 software.<br>In Mass Spectrometry based crosslinking and deuterium exchange experiments, peptides were identified using MassHunter Qualitative Analysis, version 6.0 (Agilent Technologies), Peptide Analysis Worksheet (ProteoMetrics LLC), and Pep-tideShaker, version 1.16.42, paired with SearchGUI, version 3.3.16 (CompOmics). Crosslinks were then determined using Spectrum Identification Machine (SIMXL 1.5.5.2).<br>Stopped flow kinetics data were collected using a SX-20 instrument (Applied Photophysics Inc.) and instrument-associated software provided by Applied Photophysics (Pro-Data SX ver.2.5.1852.0).<br>Analytical ultracentrifugation experiments were collected using the instrument associated software (Beckman Coulter Optima AUC Inc.).
Data analysis	Numerical data were exported and analyzed and fitted using GraphPad Prism 9 (Ver. 9.4.0 (673)) or Kaleidagraph (Ver. 4.5).<br>Circular Dichroism data were plotted using Kaleidagraph (Ver. 4.5). Steady-state fluorescence data were analyzed using GraphPad Prism 9 (Ver. 9.4.0 (673)). For The Mass Spectrometry based crosslinking and deuterium exchange experiments, peptides were identified using MassHunter Qualitative Analysis, ver-sion 6.0 (Agilent Technologies), Peptide Analysis Worksheet (ProteoMetrics LLC), and Pep-tideShaker, version 1.16.42, paired with SearchGUI, version 3.3.16 (CompOmics). Crosslinks were then determined using Spectrum Identification Machine (SIMXL 1.5.5.2). Stopped flow data were analyzed using Kaleidagraph (Ver. 4.5). C-trap data were analyzed using custom python script [Pylake API from Lumicks] and code is available upon request. Analytical ultracentrifugation experiments were analyzed using SEDFIT (Ver. 16.1c).

For manuscripts utilizing custom algorithms or software that are central to the research but not yet described in published literature, software must be made available to editors and reviewers. We strongly encourage code deposition in a community repository (e.g. GitHub). See the Nature Portfolio guidelines for submitting code & software for further information.

Data

Policy information about availability of data

All manuscripts must include a data availability statement. This statement should provide the following information, where applicable:

- Accession codes, unique identifiers, or web links for publicly available datasets
- A description of any restrictions on data availability
- For clinical datasets or third party data, please ensure that the statement adheres to our policy

All data are contained within the manuscript. Raw data and original gels are provided in the Source Data files. Plasmids used for protein overexpression are available upon request. Code for C-trap data analysis is available upon request.

Field-specific reporting

Please select the one below that is the best fit for your research. If you are not sure, read the appropriate sections before making your selection.

☒ Life sciences ☐ Behavioural & social sciences ☐ Ecological, evolutionary & environmental sciences

For a reference copy of the document with all sections, see nature.com/documents/nr-reporting-summary-flat.pdf

Life sciences study design

All studies must disclose on these points even when the disclosure is negative.

Sample size	Almost all experiments reported here were performed using purified proteins. The in vivo data were data were collected using yeast strains. For all the biochemical experiments, each experiment was repeated a minimum of three times with proteins from different preparations. Same sizes were determined based on commonly followed procedures for biophysical studies. A minimum of n=3 is required with biological samples from different protein purifications.
Data exclusions	No data were excluded from the analysis.
Replication	All experiments were replicated and repeated several times (n>3) using proteins from distinct preps.
Randomization	All the reactions were prepared and mixed independently during the repeats. These experiments were often repeated on multiple dates over the four year course of this study. Since these are targeted experiments with purified proteins, randomization is not required.
Blinding	Not applicable to this mechanistic investigation. Most experiments in this paper are done with either one, or two proteins binding to DNA. Thus, blinding is not an effective strategy - or required.

Reporting for specific materials, systems and methods

We require information from authors about some types of materials, experimental systems and methods used in many studies. Here, indicate whether each material, system or method listed is relevant to your study. If you are not sure if a list item applies to your research, read the appropriate section before selecting a response.

Materials & experimental systems

n/a	Involved in the study	
☐	☒	Antibodies
☒	☐	Eukaryotic cell lines
☒	☐	Palaeontology and archaeology
☒	☐	Animals and other organisms
☒	☐	Human research participants
☒	☐	Clinical data
☒	☐	Dual use research of concern

Methods

n/a	Involved in the study	
☒	☐	ChIP-seq
☒	☐	Flow cytometry
☒	☐	MRI-based neuroimaging

Antibodies

Antibodies used	Rfa1 antibody used was a kind gift from Dr. Steven Brill (Rutgers University) and used at 1:6000 dilution. Secondary used is Anti-rabbit HRP (used at 1:8000 dilution), Catalog number: NA934V, Lot 17320421 from Cytiva Life Sciences.
Validation	Validation for the Rfa1 antibody can be found in this publication: Brill, S. J. & Stillman, B. Replication factor-A from Saccharomyces cerevisiae is encoded by three essential genes coordinately expressed at S phase. Gene Dev 5, 1589–1600 (1991).
