# Supplementary material for: Rtt105 regulates RPA function by configurationally stapling the flexible domains
Source: Nat Commun. 2022 Sep 2;13:5152. doi: 10.1038/s41467-022-32860-6 (PMC9440123; doi:10.1038/s41467-022-32860-6)
Supplement: Supplementary file 4 — Source Data [file 41467_2022_32860_MOESM4_ESM.zip › Source Data-Uncropped Gels.pptx]

## Slide 1
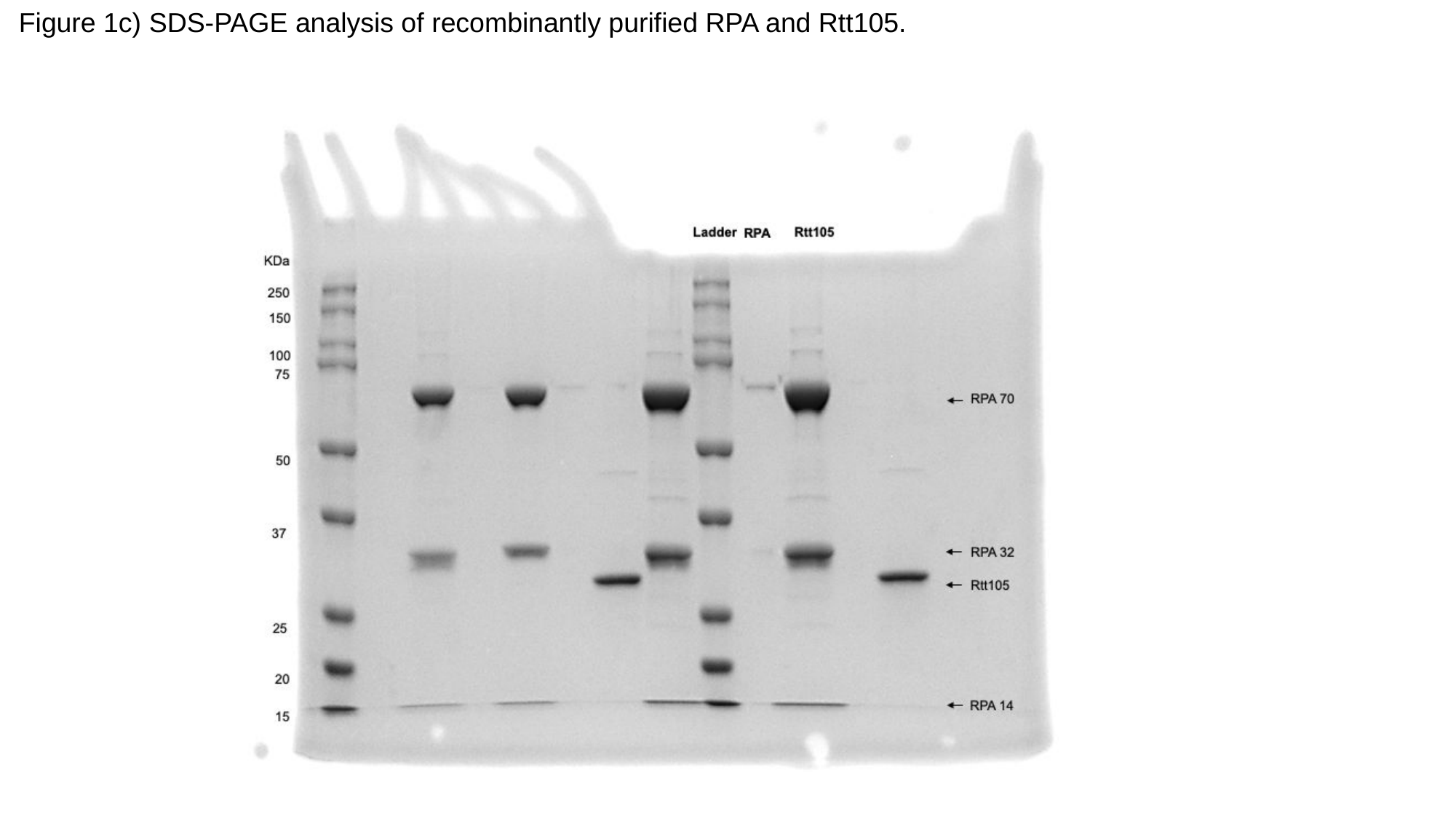

Figure 1c) SDS-PAGE analysis of recombinantly purified RPA and Rtt105.

## Slide 2
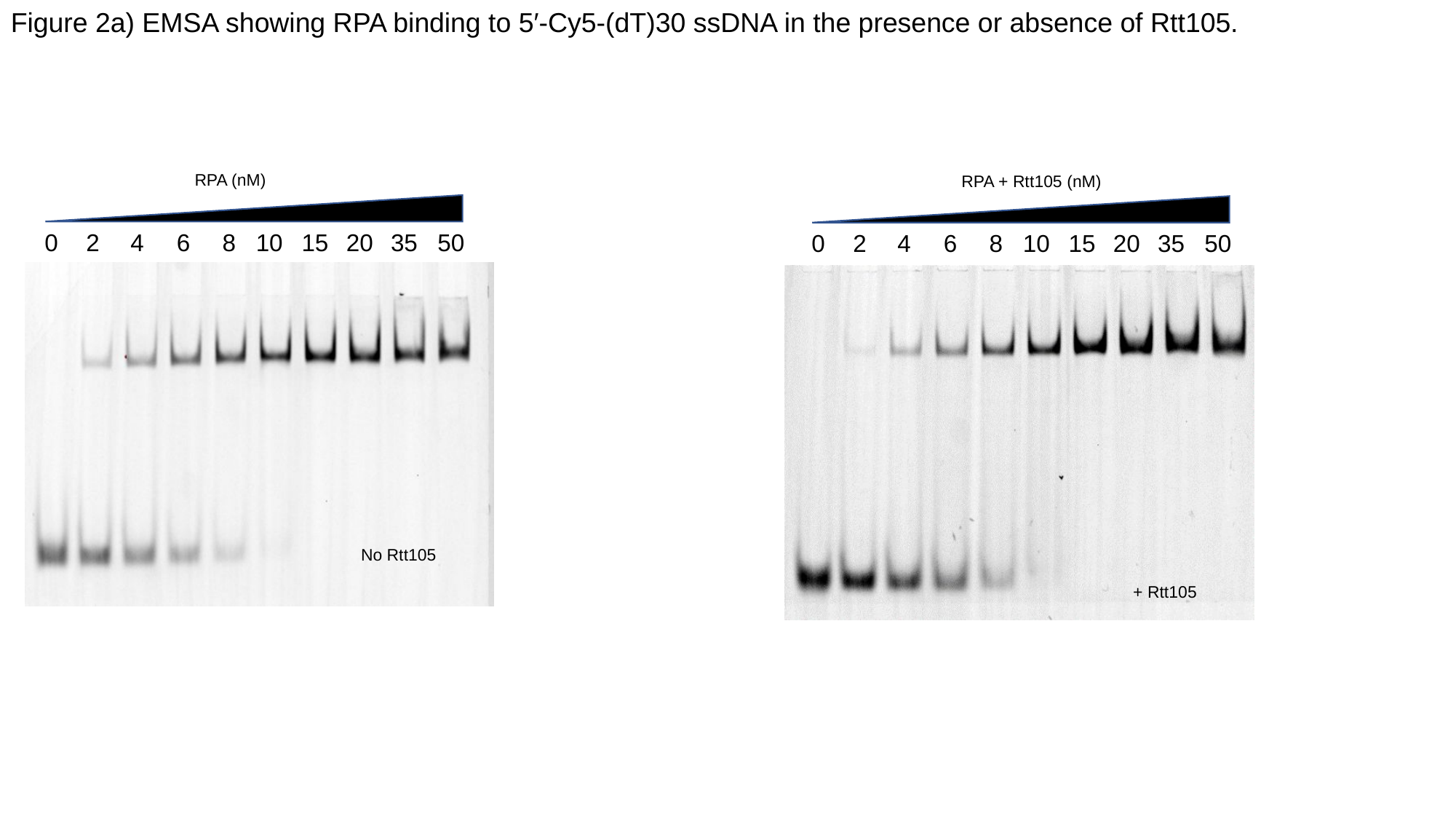

Figure 2a) EMSA showing RPA binding to 5′-Cy5-(dT)30 ssDNA in the presence or absence of Rtt105.
RPA (nM)
RPA + Rtt105 (nM)
0
2
4
6
8
10
15
20
35
50
0
2
4
6
8
10
15
20
35
50
No Rtt105
+ Rtt105

## Slide 3
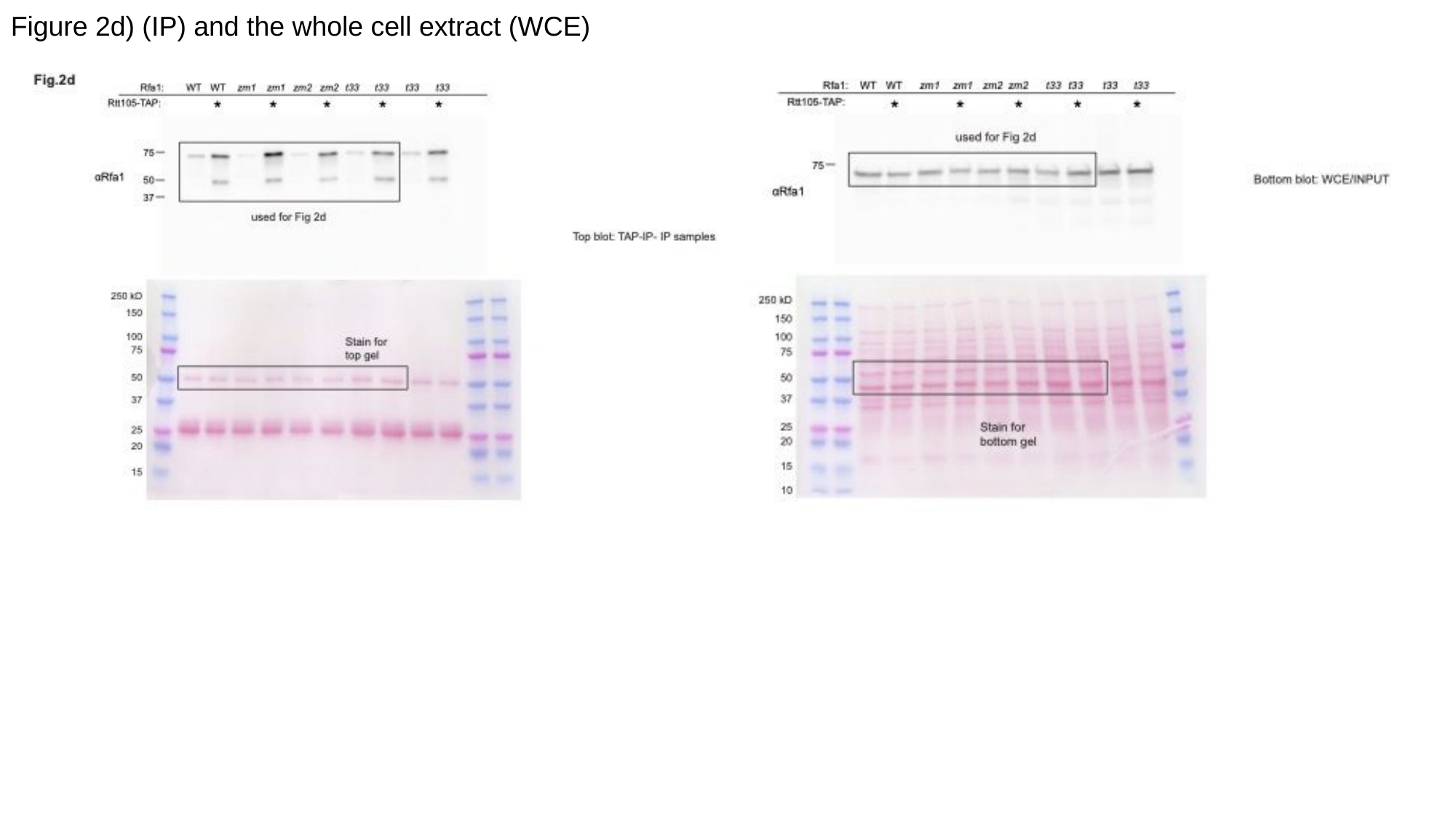

Figure 2d) (IP) and the whole cell extract (WCE)

## Slide 4
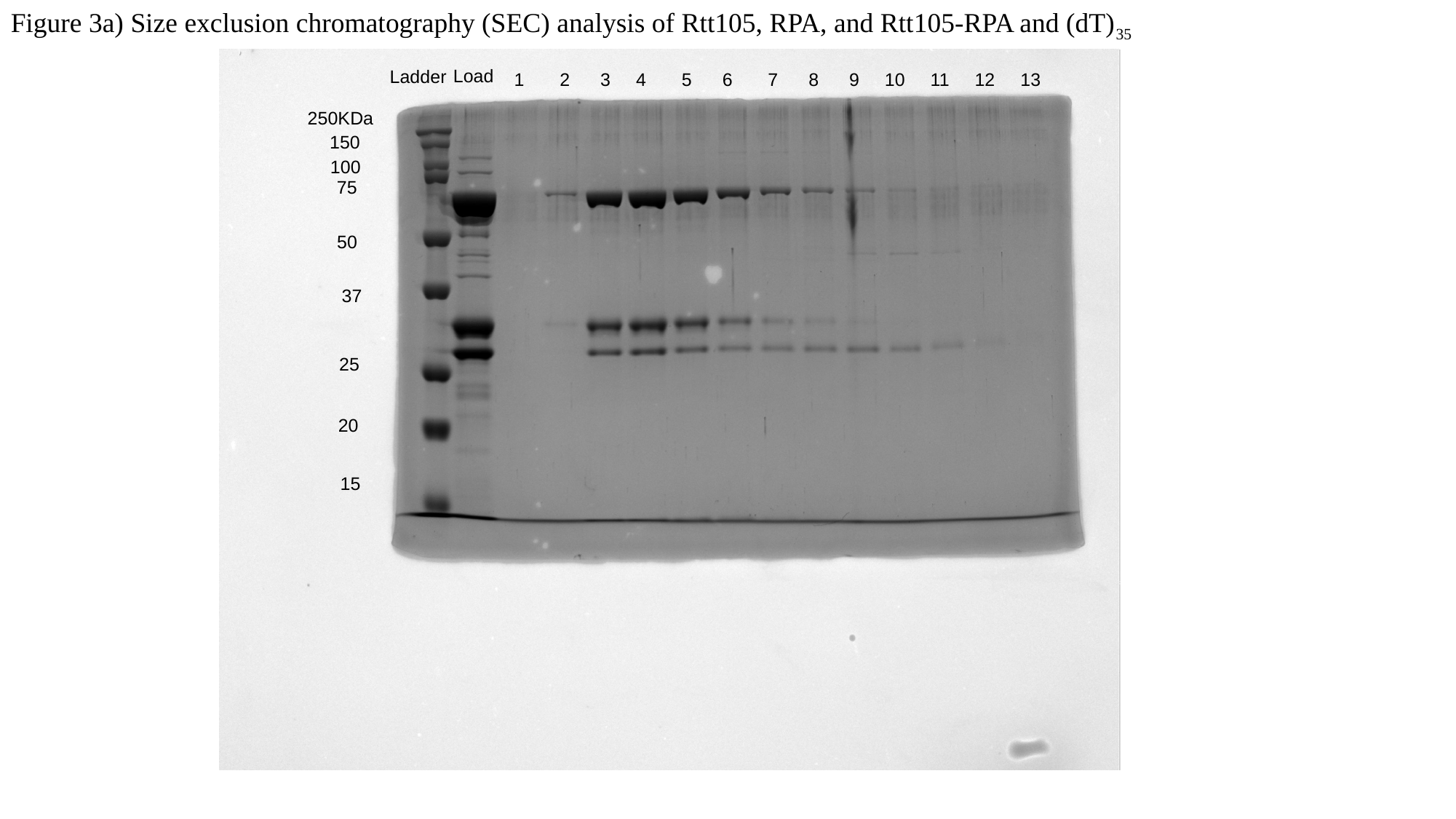

Figure 3a) Size exclusion chromatography (SEC) analysis of Rtt105, RPA, and Rtt105-RPA and (dT)35
Load
Ladder
1 2 3 4 5 6 7 8 9 10 11 12 13
250KDa
150
100
75
50
37
25
20
15

## Slide 5
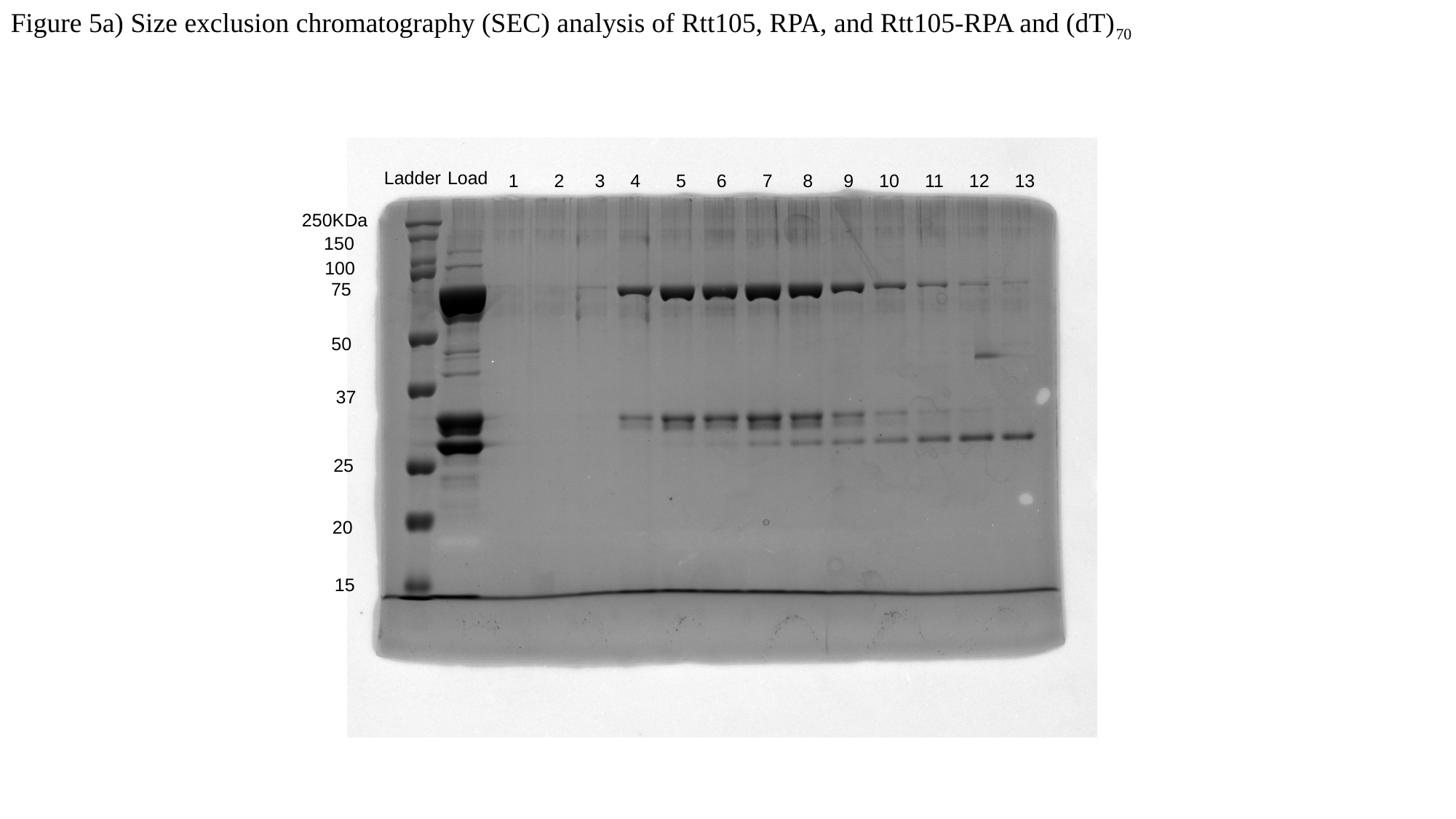

Figure 5a) Size exclusion chromatography (SEC) analysis of Rtt105, RPA, and Rtt105-RPA and (dT)70
Load
Ladder
1 2 3 4 5 6 7 8 9 10 11 12 13
250KDa
150
100
75
50
37
25
20
15
